# Precision radiotherapy for nasopharyngeal carcinoma

**DOI:** 10.1002/pro6.1219

**Published:** 2024-01-30

**Authors:** Zhenyu Zhang, Xiangzhou Chen, Taize Yuan

**Affiliations:** ^1^ Department of Radiation Oncology Guangzhou Concord Cancer Center Guangzhou Guangdong China

**Keywords:** nasopharyngeal carcinoma, precision radiotherapy, progression, target volume

## Abstract

Nasopharyngeal carcinoma(NPC) occurs frequently in Southern China, and radiotherapy is the main treatment method. At present, intensity‐modulated radiotherapy is widely used, which has improved efficacy in patients with NPC and reduced toxicity and side effects. Recently, helical tomography radiotherapy, proton radiotherapy, carbon particle radiotherapy, and other radiotherapy techniques have been used for the clinical treatment of NPC. Individualized nasopharyngeal cancer targets have also been explored. This paper reviews the research progress in radiotherapy techniques and target volume for NPC

## Graphical abstract

Nasopharyngeal carcinoma occurs frequently in Southern China, and radiotherapy is the main treatment method. At present, intensity modulated radiotherapy (IMRT) has been widely used, which has improved the efficacy of nasopharyngeal carcinoma patients and reduced the toxicity and side effects. In recent years, helical tomography radiotherapy, proton radiotherapy, carbon particle radiotherapy and other radiotherapy techniques have been used in the clinical treatment of nasopharyngeal carcinoma. This paper reviewed the progression and clinical applications of theses new radiotherapy technique on nasopharyngeal carcinoma.

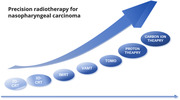

Nasopharyngeal carcinoma (NPC) has a unique geographical distribution compared with other malignant tumors of the head and neck. Although it is rare globally, NPC is an endemic disease in Southern China, with incidence rates ranging from 15 to 50 per 100,000.^1^, ^2^ Because the nasopharynx is close to the skull base and middle cranial fossa, it is easy for the tumor cells of NPC to invade the cavernous sinus and optic pathway. Therefore, they are unsuitable for surgery. Additionally, the pathology of NPC is mainly “non‐keratinizing cancer,” which is World Health Organization group III and sensitive to irradiation. Therefore, radiotherapy is the first choice of treatment for patients who are newly diagnosed with nasopharyngeal carcinoma without metastasis.^3^ Moreover, intensity‐modulated radiotherapy (IMRT) is widely used. Helical tomotherapy (HT), proton radiotherapy, and carbon particle radiotherapy have also been used to treat NPC in recent years, and their efficacy and related toxic reactions have been reported one after another. In the era of precision radiotherapy, delineating individual target volumes for different NPC patients can reduce irradiated volumes as much as possible while ensuring efficacy, which helps improve the quality of life of patients. This paper reviewed the recent advances in radiotherapy techniques and target volumes for NPC.

## 1 IMRT

Since the beginning of the 21st century, IMRT has been used in China. Compared with two‐dimensional conventional radiotherapy (2D‐CRT), IMRT has a significant advantage in terms of dosimetric distribution. It has achieved higher radiation doses to the tumor target while reducing exposure to adjacent organs. Furthermore, in 2012, Peng et al.^4^ reported the result of the only prospective study comparing the efficacy and toxicity of IMRT and 2D‐CRT in 616 patients who are newly diagnosed with non‐metastatic nasopharyngeal carcinoma. This study revealed that, compared with the 2D‐CRT group, the 5‐year local control rates of T3 and T4 patients in the IMRT group increased by 11% (91% vs. 80%) and 19.3% (81.5% vs. 62.2%), respectively, and the 5‐year overall survival (OS) increased by 12.5% (79.6% vs. 67.1%, *P* = 0.001). However, the incidence of late toxic side effects such as temporal lobe neuropathy (13.1% vs. 21%), cranial nerve palsy (3.9% vs. 8.7%), xerostomia (39% vs. 99%), hearing loss (25% vs. 84%), trismus (3.3% vs. 13.9%), and neck fibrosis (2.3% vs. 11.3%) significantly decreased. In 2020, Liao et al.^5^ retrospectively reported the efficacy and toxicity of IMRT in the treatment of advanced NPC without distant metastasis and skull base bone invasion compared to 2D‐CRT. Additionally, compared to 2D‐CRT, IMRT significantly improved four‐year local region‐relapse‐free survival rate (76.5% vs. 87.5%, *P* =  0.003), disease‐free survical (DFS) (57.3% vs. 73.3%, *P* <0.001), and OS (65.6% vs. 81.8%, *P* < 0.001) in patients with NPC with severe skull‐base invasion (*n* = 466). In the IMRT group, lower incidences of mucositis (39% vs. 51.1%), xerostomia(27.5% vs. 58.4%), trismus (< 1 cm) (3.9% vs. 9.9%), and temporal lobe necrosis (15% vs. 29.2%)were found than in the 2D‐CRT group. Furthermore, IMRT can significantly improve local control and reduce radiotherapy‐induced toxicities in patients with locally advanced NPC.

In terms of the differences between IMRT and 3D‐CRT for NPC, a prospective study was published by Fang et al.^6^ in 2008 that compared the efficacy and quality of life of the two radiotherapy technologies in 203 patients with non‐metastatic NPC. The 3‐year locoregional control, metastasis‐free survival, and OS rates were 84.2%, 82.6%, and 85.4% in the IMRT group, respectively, compared with 84.8%, 76.7%, and 81.7% in the 3D‐CRT group (*P* > 0.05). However, IMRT significantly enhanced the patients’ global quality of life, reduced fatigue, improved taste and smell perception, lessened dry mouth, and reduced discomfort at the third month after radiotherapy. At other time points, there were no significant differences in most scales between the two groups. In 2015, Sung et al.^7^ retrospectively analyzed the efficacy of IMRT (*n* = 497) compared to 3D‐CRT (*n* = 390) in the treatment of NPC, and found no statistical difference in the 5‐year OS between the two groups (73.6% vs. 76.7%, *P* > 0.05). However, in the T3‐4 subgroup, IMRT was associated with a significantly better 5‐year OS than 3D‐CRT (70.7% vs. 57.8%, *P* = 0.011).

## 2 VOLUMETRIC‐MODULATED ARC THERAPY

Volumetric‐modulated arc therapy (VMAT) is a new technique based on conventional IMRT, which can dynamically alter the dose delivery of various gantry arcs as the gantry rotates around a patient. The gantry can simultaneously modulate the position, dose rate, and gantry velocity of the multiblade collimator. Moreover, compared to static IMRT technology, VMAT does not require a limited number of fixed gantry angles and has greater flexibility because it delivers radiation therapy from all beam directions. Additionally, it can complete the entire treatment with a 360° rotation of the gantry frame and shorten the time of each fraction for patients receiving the same dose of irradiation. This technique can effectively alleviate the situation in large medical centers with several patients and long treatment times.

Several studies have compared the dosimetry and efficacy of IMRT and VMAT for the treatment of NPC. He et al.^8^ compared the tumor target coverage and organs at risk (OAR) limit between IMRT and VMRT in patients with NPC. This study revealed that the doses to the planning target volume of the gross nasopharynx (PGTVnx), 95% of the PGTVnx (D_95_), and 98% of the PGTVnx (D_98_) were significantly higher with VMAT than with IMRT. However, the maximum radiation dose to the brain stem, spinal cord, and optic nerve, as well as the incidence of late side effects, such as ototoxicity(16.4% vs. 25.1%), trismus(3% vs.7.2%), and temporal lobe necrosis(3.3% vs. 6.9%), were lower in the VMAT group than in the IMRT group. Chen et al.^9^ reported the differences between the two radiotherapy techniques in the treatment of NPC, and found that VMAT can also decrease late toxicity for ototoxicity, spasm, and temporal lobe injury. Compared with IMRT (1160 ± 204s), VMAT (363 ± 162s) shortened the treatment time by 69%. However, the 2‐year local recurrence‐free survival, local recurrence‐free survival, distant metastasis‐free survival, disease‐free survival, and OS rates were similar between the two groups. Furthermore, another study on NPC published by Huang et al.^10^ in 2020 found no significant differences in the 3‐year local control rate, distant metastases‐free survival rate, and OS rates between the VMAT group (*n* = 66) and the IMRT group (*n* = 74).

Compared with IMRT, VMAT can effectively shorten the treatment time for patients with NPC with similar outcomes. However, whether VMAT can effectively decrease the radiation dose to OARs remains controversial and requires further investigation.

## 3 TOMOTHERAPY

HT is an emerging IMRT technology that installs a 6‐MV linear accelerator on a ring frame around the accelerator couch 360. When the irradiation target area rotates, the treatment couch advances axially through the center of the stand. The equipment and treatment cost of the TOMO are higher than that of the linear accelerator, treatment time is longer than that of the linear accelerator, and it can be further extended by adjusting parameters such as gate width, pitch, and modulation factor. However, TOMO is superior to conventional accelerators in terms of dose uniformity, a steep dose gradient, and protection of normal organs, which is also reflected in the treatment of head and neck tumors.

In 2021, Lu et al.^11^ compared the dosimetric parameters of different radiotherapy plans (HT, VMAT, and fixed‐field IMRT) for 15 patients with locally advanced NPC, and found that HT plans significantly improved the mean conformity index and homogeneity index compared to VMAT and fixed‐field‐IMRT plans. The HT plans decreased the maximum doses to OARs including the brainstem, spinal cord, and optic nerves. Moreover, the volume of normal tissue irradiated with high doses, such as V30 of the parotid glands, was significantly reduced in the HT plans. Du et al.^12^ summarized the outcomes for 190 patients with NPC treated with TOMO. The 3‐year regional recurrence‐free survival rate, local recurrence‐free survival rate, distant metastasis‐free survival rate and OS rate of patients receiving TOMO radiotherapy were 98.2%, 96.1%, 92.0% and 86.3% respectively. This study indicated that TOMO radiotherapy demonstrated very low toxicity and good outcomes in patients with NPC. Another study retrospectively compared the efficacy of TOMO and IMRT in the treatment of pharyngeal cancers and found no significant difference in the 5‐year OS (79% vs. 74%, *P* = 0.15) between the two techniques.^13^ However, in the present retrospective study, 101 patients in the TOMO and IMRT groups had non‐nasopharyngeal cancer. Therefore, the effectiveness of TOMO and IMRT requires further investigations.

## 4 PROTON RADIOTHERAPY

Proton therapy is a new technology that uses the unique “Bragg peak” characteristics of the proton beam to treat tumors. It has been widely used in Europe, the United States, and other developed countries. As photons enter the human body to a certain depth, their energy gradually decreases, and the dose intensity is greatly reduced when they reach the focus. However, owing to its special “Bragg peak” characteristics, proton therapy keeps the dose basically stable after entering the human body, until the radiation reaches the focus and releases all energy at the peak, realizing “directional explosion” to the tumor, and rapidly decreases after passing the focus and decays to zero in a very short distance.^14^ In recent years, intensity‐modulated proton therapy (IMPT) has been developed to reduce the radiation dose to normal tissues in front of a tumor, and the physical advantages of proton beams have been further improved. Proton therapy has more obvious physical advantages than photon therapy. It can significantly reduce the radiation dose to normal tissues around the tumor while keeping the tumor dose unchanged to reduce the adverse reactions caused by radiotherapy, and also provide the possibility for tumor dose escalation.^15^ Furthermore, unlike photons, the major challenge of proton therapy is uncertainty. In head and neck tumors, the position of the Bragg peak can shift owing to positioning errors, artifacts, weight loss, and tumor regression; therefore, proton therapy is very demanding.

The MD Anderson Cancer Center reported 10 patients with NPC treated with IMPT, nine of whom had a photon‐based IMRT plan for comparison.^16^ The mean doses of the 29 adjacent OARs were compared dosimetrically. Compared to the photon‐based IMRT plan, 13 OARs received a lower mean dose with the proton‐based plans. This study demonstrated that IMPT has dosimetric advantages over IMRT. Further studies are required to determine whether these factors translate into lower toxicity and/or higher disease control. In 2020, JIRI et al.^17^ evaluated the outcomes and toxicities in 40 patients with NPC treated with IMPT. Among the 40 patients, 80% (32/40) were in stages III‐IVB, and the median dose was 74 GyE (70–76 GyE) in 37 fractions (35–38). Concomitant chemotherapy was administered in 34 patients. The 2‐year OS, disease control, and local control rates were 80%, 75%, and 84%, respectively. Additionally, the incidence rates of grade 3 and above acute radiation skin injury, oral mucositis, and dysphagia were 14%, 7%, and 9.3%, respectively, and 5% of the patients had grade 3 or higher late toxicity and side effects (dysphagia and brain necrosis). These results suggest that IMPT is feasible for patients with NPC with mild acute toxicity. A propensity score‐matched analysis^18^ on IMPT or IMRT for NPC patients showed IMPT for non‐metastatic NPC was associated with significantly reduced ≥grade 3 acute toxicity burden in comparison with IMRT (10.7% vs. 22.4%). Grade 3 or higher late complications were 3.8% in IMPT and 16.3% in IMRT, respectively. The 2‐year progression‐free survival (PFS) and OS rates were 95.7% and 100% in the IMPT group and 76.7% and 94.1% in IMRT, respectively. The 2‐year local regional recurrence‐free survival tended to be significantly different between the two groups (100% in IMPT vs. 86.2% in IMRT, *P* = 0.08). Lee et al.^19^ systematically analyzed nine articles (369 patients) on proton therapy for NPC and showed that IMPT was associated with less acute toxicity and late complications compared to IMRT in 2‐year local recurrence‐free survival, with good outcomes.

Proton radiotherapy has also been reported in patients with recurrent NPC.^20^ Seventeen patients with recurrent NPC were treated with proton radiotherapy at a median dose of 60 relative biological effectiveness (RBE) (range 30.6–66Gy). The OS and local control rates at 18 months were 54.4% and 66.6%, respectively. No grade 3 or above acute side effects were observed. However, 23.5% of patients had advanced grade 3 or higher side effects. Serious toxic side effects of radiotherapy for recurrent NPC include massive nasopharyngeal hemorrhage and temporal lobe necrosis, which are also important causes of death. Therefore, it is believed that if proton radiotherapy can effectively reduce these risks, it will help improve the quality of life for patients with recurrent NPC; moreover, its long‐term efficacy of this treatment approach needs further observation.

## 5 CARBON‐ION RADIOTHERAPY

Carbon‐ion radiotherapy and proton therapy are particle radiotherapies, and both have the physical properties of the Bragg peak, which allows for higher dose distribution accuracy than photon‐based radiotherapy and provides better protection of the normal tissue around the tumor than photon beams. However, in contrast to protons, carbon‐ion beams have a higher linear energy transfer and RBE, and the killing effect on cells is more obvious than that of protons and photons.^21^ At present, there are limited studies on carbon‐ion treatment of NPC.

Wang et al.^22^ compared the radiotherapy plans of intensity‐modulated carbon‐ion radiation therapy (IMCT) and IMRT for patients with NPC and found that both plans met the clinical prescription dose requirements and showed satisfactory coverage of the target without significant differences. The IMCT plans were superior to the IMRT plans in the protection of OARs, including the brain stem, spinal cord, parotid gland, optic chiasm, eyeball, lens, temporal lobe, and inner ear. However, whether this dosimetric advantage translates into reduced toxicity and improved quality of life remains to be further investigated. In 2021, Hu et al.^23^ reported the clinical efficacy in 69 patients with non‐metastatic NPC who received IMRT + carbon‐ion radiotherapy (CIRT)‐enhanced therapy at the Shanghai Proton Heavy Ion Center in 2021. Of these patients, 74% had locally advanced disease (stage III/IV). The median follow‐up was 31.9 months, and the 3‐year OS, PFS, local control rate, and remote control rate were 94.9%, 85.2%, 96.9%, 98.4%, and 89.7%, respectively. Severe acute toxicity from radiotherapy was observed in only two patients (dermatitis). This study showed that a combination of IMRT and CIRT boost therapy provided excellent disease control and a favorable toxicity profile for patients with non‐metastatic NPC. Hu et al.^24^ also reported the efficacy of carbon‐ion radiotherapy in 206 patients with locally recurrent NPC, with prescription dose of 50–69 GyE (2.0‐3.0 GyE/f) and median follow‐up of 22.8 months. The 2‐year OS, local, regional, and distant metastasis control rates were 83.7%, 58.0%, 87.3%, and 94.7%, respectively. No acute toxicity or grade 3 or higher adverse effects were observed during radiotherapy. The incidence rates of grade 3 or above advanced toxic and side effects were 0.97% temporal lobe necrosis and 0.49% cranial nerve paralysis. Hearing loss was 1.46%, xerostomia was 0.49%, and mucosal necrosis was 16.02%, indicating that salvage therapy with CIRT was effective in patients with local recurrence NPC with acceptable toxicity.

## 6 TARGET VOLUME PROGRESS

With the improvement in the prognosis of patients with NPC, improving their quality of life has become an increasingly important issue. It is believed that by narrowing the target volume without affecting efficacy, the toxic side effects of radiotherapy can be reduced and the quality of life can be improved. In recent years, some clinical research centers have conducted relevant studies on the target volume of NPC.

In 2018, Yang et al.^25^ reported a phase III clinical trial comparing the efficacy and quality of life of irradiation therapy based on GTVnx, clinical target volume (CTV)1, and CTV2 deletions before and after neoadjuvant chemotherapy. Totally 97 cases were treated with the pre‐neoadjuvant chemotherapy target volume (group A). A total of 115 patients were treated with the target volume after neoadjuvant chemotherapy (group B). The results showed that the volumes of GTVnx and CTV1 in group B were significantly smaller than those in group A, and the volume of irradiation received by some of the OARs (including the temporal lobe, optic nerve, lens, and parotid gland) was also significantly smaller than that in group A. Survival analysis showed no significant difference in the 3‐year OS between the two groups; patients in group B had no increased risk of local recurrence, and their cognitive function was significantly better than that of group A two years after the end of treatment. Reducing the target volume is safe and feasible for patients who experience tumor shrinkage after neoadjuvant chemotherapy. In 2022, Tang et al.^26^ reported a clinical trial that addressed whether elective ipsilateral upper neck irradiation (UNI) provides a similar regional relapse‐free survival rate as standard whole‐neck irradiation (WNI). A total of 446 patients with N0‐N1 NPC were enrolled and randomly assigned in a 1:1 ratio. Patients with N0 and retropharyngeal lymph node metastases received bilateral UNI; patients with N1 received negative UNI; and the control group received WNI. The results showed that the three‐year regional recurrence, OS, distant metastasis, and local recurrence rates were not significantly different between the two groups. However, sparing the skin of the lower neck, thyroid glands, and pharyngeal constrictors from irradiation reduced the toxic side effects of radiotherapy, including neck tissue damage, hypothyroidism, and dysphagia, and the quality of life of the patients was also significantly improved. In 2023, Mao et al.^27^ reported the clinical outcomes of medial retropharyngeal lymph node (MRLN)‐sparing radiotherapy compared to standard radiotherapy (medial and lateral retropharyngeal lymph node groups). In total, 568 patients were recruited including 285 in the sparing group and 283 in the standard group. The results showed that the 3‐year local relapse‐free survival rate in the MRLN‐sparing radiotherapy group was not inferior to that in the standard radiotherapy group. Similarly, the 3‐year OS, regional recurrence‐free survival, and distant metastasis‐free survival rates were similar between the two groups. In terms of toxicity and quality of life, the incidence of radiation‐related toxicity, including acute mucositis, acute dysphagia, weight loss, and late dysphagia, was lower in the sparing group. In addition, global health status, role functioning, fatigue, and swallowing in the sparing group were better than in the standard group.

## 7 CONCLUSION

In the era of IMRT, the therapeutic efficacy in patients with NPC has improved, whereas the toxicities of radiotherapy have reduced. IMRT, VMAT, and TOMO have similar efficacy, but whether they can effectively reduce the radiation dose to the OARs and whether VMAT can effectively shorten the treatment time require further investigation. Currently, studies on proton and carbon‐ion radiotherapy for the treatment of newly diagnosed NPC are limited, with small sample sizes. The potential of these theapies to reduce short‐ and long‐term toxicity in patients awaits confirmation from prospective studies with larger sample sizes. Furthermore, in the era of precision radiotherapy, it is important to delineate individualized target volumes for patients based on tumor volume after neoadjuvant chemotherapy or lymph node status .

## CONFLICT OF INTEREST STATEMENT

The authors declare no conflicts of interest.
